# Use of Tacrolimus and Voclosporin as Induction Therapy in Lupus Nephritis: A Focused Systematic Review and Meta-Analysis of Randomized Evidence

**DOI:** 10.3390/jcm15197607

**Published:** 2026-09-30

**Authors:** Urvi Zala, Rushi Patel, Jacob Thompson, Anil Regmi

**Affiliations:** 1 Department of Internal Medicine, Loyola Medicine MacNeal Hospital, Berwyn, IL 60402, USA; 2 Department of Internal Medicine, Baptist Hospitals of Southeast Texas, Beaumont, TX 77701, USA; 3 Paul L. Foster School of Medicine, Texas Tech University Health Sciences Center El Paso, El Paso, TX 79905, USA; 4 Department of Internal Medicine, Parkview Health, Fort Wayne, IN 46845, USA

**Keywords:** lupus nephritis, calcineurin inhibitors, tacrolimus, voclosporin, meta-analysis

## Abstract

**Background**: Lupus nephritis (LN) remains a major cause of morbidity among patients with systemic lupus erythematosus, and treatment resistance continues to pose clinical challenges. Calcineurin inhibitors (CNIs), including tacrolimus (TAC) and voclosporin (VCS), are used in induction regimens, but randomized evidence for the two agents arises from clinically different trial designs. This systematic review and meta-analysis separately quantifies randomized evidence for TAC-based and VCS-based induction regimens. **Methods**: Following PRISMA guidance, the literature search was updated to September 2026. Eligible studies were randomized controlled trials enrolling adults with biopsy-proven active class III, IV, or V LN and were required to report complete and/or partial renal-response data in a form permitting extraction or calculation of an effect estimate. Study inclusion was not based on whether the treatment effect was favorable or statistically significant. Complete renal response was the prespecified primary outcome for quantitative synthesis. Separate random-effects meta-analyses were performed for TAC-based and VCS-based regimens. Because the two evidence sets did not share a sufficiently comparable common comparator, no formal TAC-versus-VCS Bucher comparison was retained. **Results**: Five randomized controlled trials were included in the quantitative synthesis: three TAC trials and two VCS trials. TAC-based regimens increased complete renal response relative to their respective comparators (RR, 1.36; 95% CI, 1.00–1.86), although heterogeneity was substantial (I^2^ = 73%) and sensitivity analyses showed that the pooled result was influenced by the multitarget TAC + MMF trial. VCS added to MMF and low-dose glucocorticoids increased complete renal response versus the placebo with the same background therapy (RR, 1.78; 95% CI, 1.35–2.34; I^2^ = 0%). Safety outcomes were summarized descriptively because definitions and reporting differed across trials. **Conclusions**: TAC-based and VCS-based induction regimens each showed benefits within their respective randomized trial settings. Differences in background therapy, comparators, study populations, and outcome definitions preclude a reliable indirect efficacy comparison between TAC and VCS. Head-to-head randomized trials using standardized background therapy and outcomes are needed.

## 1. Introduction

Lupus nephritis (LN) is one of the most severe manifestations of systemic lupus erythematosus (SLE), a chronic autoimmune disease characterized by dysregulated immune activation, autoantibody production, and immune complex deposition [1]. It occurs in approximately 40–60% of patients with SLE and represents a major contributor to morbidity and mortality worldwide [2]. The pathogenesis of LN is multifaceted, involving the deposition of circulating immune complexes in the glomerular basement membrane, activation of the complement cascade, and recruitment of inflammatory cells, culminating in glomerular and tubulointerstitial injury [3]. Over time, this inflammatory environment promotes fibrosis, proteinuria, and progressive renal dysfunction, ultimately increasing the risk of chronic kidney disease (CKD) and end-stage renal disease (ESRD) [4]. The clinical spectrum of LN is diverse, ranging from asymptomatic urinary abnormalities to rapidly progressive glomerulonephritis [5]. Histopathological classification, defined by the ISN/RPS criteria, provides prognostic information and guides therapeutic decisions, with class III (focal proliferative) and class IV (diffuse proliferative) forms being the most aggressive and associated with poor renal outcomes [6]. Despite advances in diagnosis and therapy, LN remains a leading cause of death and disability in patients with SLE, underscoring the need for effective, durable, and well-tolerated treatment strategies.

Current treatment strategies for active LN are built upon a combination of immunosuppressive agents designed to induce remission and prevent flares while minimizing long-term organ damage. Conventionally, glucocorticoids have been the cornerstone of therapy, exerting broad anti-inflammatory effects by suppressing pro-inflammatory cytokines and immune cell proliferation [7]. However, their long-term use is limited by substantial toxicity, including increased risks of infection, cardiovascular disease, osteoporosis, and metabolic derangements [8]. As a result, steroid-sparing regimens have become a major focus of modern LN management. Mycophenolate mofetil (MMF) has emerged as a first-line immunosuppressant for induction and maintenance therapy, supported by multiple randomized controlled trials demonstrating efficacy comparable to cyclophosphamide with a more favorable safety profile [9]. Cyclophosphamide, once the gold standard for proliferative LN, remains an important option, particularly for severe disease, but its use is tempered by risks such as infertility, cytopenias, and malignancy [10]. There has been a major change in the approach to managing active LN, as outlined by the 2024 ACR guidelines, which includes aggressive immunosuppressive regimens with the introduction of triple immunosuppressive therapy using calcineurin inhibitors (CNIs) or belimumab (BEL) on a background of mycophenolate acid analogs (MPAA) and glucocorticoids (GC), with an emphasis on early glucocorticoid tapering to minimize long-term toxicity [11].

Calcineurin inhibitors (CNIs) have emerged as an important therapeutic class in LN due to their dual mechanism of action, involving the suppression of T-cell activation through inhibition of calcineurin–NFAT signaling and stabilization of podocytes to reduce proteinuria independent of immune modulation [12]. Tacrolimus (TAC), long used in transplantation and increasingly applied in LN, has shown efficacy both alone and in combination with MMF and glucocorticoids, particularly in Asian populations where multitarget regimens are common, though concerns about dosing variability, nephrotoxicity, and metabolic effects persist [13,14]. Voclosporin (VCS), a structurally modified cyclosporine analog, offers improved binding affinity, metabolic stability, and pharmacokinetics, eliminating the need for routine drug monitoring [15]. Recent randomized controlled trials have demonstrated that VCS, added to MMF and low-dose glucocorticoids, achieved significantly higher renal response rates with an acceptable safety profile, leading to its approval as a novel therapeutic option [16,17]. Together, these agents highlight the expanding role of CNIs in LN, though differences in efficacy, safety, and long-term outcomes remain to be clarified.

Despite the growing evidence base, important gaps remain in understanding how TAC-based and VCS-based induction regimens perform in LN. No head-to-head randomized controlled trial has directly compared TAC with VCS. In addition, the TAC trials used heterogeneous regimens and comparators, including TAC plus glucocorticoids versus MMF plus glucocorticoids, TAC + MMF multitarget therapy versus intravenous cyclophosphamide, and TAC plus glucocorticoids versus intravenous cyclophosphamide. By contrast, the VCS trials evaluated VCS versus placebo on a shared background of MMF and rapidly tapered low-dose glucocorticoids. These differences limit cross-trial comparability and make a formal adjusted indirect comparison methodologically uncertain.

Accordingly, this study aimed to separately synthesize randomized evidence for TAC-based and VCS-based induction regimens in adults with active LN, with complete renal response as the primary pooled efficacy outcome. A secondary objective was to examine the clinical and methodological heterogeneity of the TAC evidence, including the distinction between TAC dual therapy and TAC + MMF multitarget therapy, and to summarize trial-reported safety outcomes. Comparative conclusions between TAC and VCS were intentionally avoided because the available trial sets do not form a sufficiently comparable common-comparator network.

## 2. Methods

### 2.1. Eligibility Criteria

This systematic review and meta-analysis was conducted in accordance with the Preferred Reporting Items for Systematic Reviews and Meta-Analyses (PRISMA) guidelines (provided in Supplementary File S1). Eligible studies were randomized controlled trials were performed evaluating TAC- or VCS-based induction therapy in adults (≥18 years) with biopsy-proven active LN (ISN/RPS class III, IV, or V). Because the primary objective of this focused review was quantitative synthesis of renal response, trials were required to report complete and/or partial renal-response outcomes in a form permitting extraction or calculation of an effect estimate. Eligibility was not determined by whether the renal-response result favored the CNI or achieved statistical significance. Trials could compare the CNI-based regimen with MMF, cyclophosphamide, or a placebo plus protocol-specified background therapy. Because the TAC trials differed in whether TAC was used as dual therapy or as TAC + MMF multitarget therapy, these regimens were pooled only as a broad “TAC-based regimen” analysis and were examined separately in sensitivity analyses. This review was not prospectively registered.

Exclusion criteria included non-randomized or uncontrolled studies, reviews, case reports, editorials, commentaries, conference abstracts, animal or in vitro studies, mixed autoimmune nephropathies without stratified LN data, maintenance-only CNI therapy, studies not meeting the prespecified adult biopsy-confirmed LN population, and reports that did not provide sufficient renal-response data to calculate or extract an effect estimate. The latter criterion reflected the prespecified quantitative objective of the review rather than the direction or statistical significance of the treatment effect. Only full-text articles published in English were considered, with no restrictions on geographical location or sample size.

### 2.2. Information Sources

A comprehensive electronic search was conducted using ClinicalTrials.gov, PubMed, ScienceDirect, PubMed Central (PMC), Embase, Web of Science, the Cochrane Library, and Google Scholar. The original search was conducted through June 2025 and was subsequently updated through September 2026 to resolve the search-date discrepancy. The updated search identified an additional randomized three-arm trial published in 2025 comparing cyclophosphamide, MMF, and TAC; it was excluded because the study enrolled participants aged ≥10 years and permitted clinical rather than mandatory biopsy-proven LN, which did not meet the prespecified adult biopsy-confirmed eligibility criteria. No additional study meeting all eligibility criteria was identified, so the quantitative analytic set remained unchanged at five RCTs. Reference lists of included studies and relevant reviews were also screened.

Gray literature sources such as unpublished dissertations, preprints, or government reports were excluded to maintain methodological transparency and data reliability. No restrictions were placed on the study setting or participant demographics.

### 2.3. Search Strategy and Selection Process

The search strategy incorporated controlled vocabulary and free-text terms related to lupus nephritis and calcineurin inhibitors. Core terms included “lupus nephritis,” “systemic lupus erythematosus,” “tacrolimus,” “voclosporin,” “calcineurin inhibitor,” “renal response,” “complete renal response,” “complete remission,” “partial remission,” and “randomized controlled trial.” Search results were screened independently by two reviewers. The September 2026 update used the same conceptual eligibility framework as the original search. 

All retrieved citations were screened independently by two reviewers. Titles and abstracts were first reviewed for relevance, followed by full-text assessment of potentially eligible studies. Reviewers resolved discrepancies through discussion and consensus. A PRISMA-compliant flow diagram was used to document the number of records identified, screened, excluded, and included in the final synthesis.

### 2.4. Inclusion Criteria

Eligible studies included randomized controlled trials enrolling adults aged ≥18 years with biopsy-proven active lupus nephritis (ISN/RPS Class III, IV, or V) undergoing induction therapy. Trials were required to evaluate a tacrolimus- or voclosporin-based induction regimen against an eligible comparator such as MMF, intravenous cyclophosphamide, or a placebo with protocol-specified background therapy and to report quantifiable complete and/or partial renal-response outcomes. Study inclusion did not depend on whether the efficacy result favored TAC/VCS or reached statistical significance. Complete renal response/remission was prespecified as the primary endpoint for quantitative synthesis.

### 2.5. Data Collection

Data were independently extracted by two investigators using a standardized form. Extracted variables included randomized sample size, efficacy-analysis population, safety population, intervention and comparator regimens, background immunosuppression, CNI dosing, follow-up duration, trial-specific complete renal response definition, event counts, and safety outcomes. The primary pooled endpoint was complete renal response/remission at the prespecified induction endpoint. Complete and partial response were not combined in the primary meta-analysis.

For each efficacy analysis, the exact numerator and denominator used in the published trial analysis were recorded. Mok et al. [18] contributed 46/74 complete responses with TAC and 45/76 with MMF; Liu et al. [19] contributed 83/181 responses with TAC + MMF multitarget therapy and 46/181 with intravenous cyclophosphamide; Zheng et al. [20] contributed 70/141 responses with TAC and 45/124 with intravenous cyclophosphamide from the reported per-protocol efficacy set; AURA-LV [21] contributed 29/89 responses with low-dose VCS 23.7 mg twice daily and 17/88 with placebo; and AURORA-1 [22] contributed 73/179 responses with VCS and 40/178 with placebo. Safety analyses used the safety populations reported by each trial and were not assumed to share the same denominator as the efficacy analyses.

### 2.6. Study Risk of Bias Assessment

The methodological quality of all included RCTs was assessed using the Cochrane Risk-of-Bias 2.0 (RoB 2) tool. Domains evaluated included the randomization process, deviations from intended interventions, missing outcome data, measurement of outcomes, and selective reporting. Each domain was rated as having “low concerns,” “some concerns,” or “high concerns” of bias. Disagreements were resolved by consensus. The overall risk-of-bias summary was presented graphically to provide an overview of methodological quality across studies.

### 2.7. Synthesis Methods

Separate meta-analyses were conducted for TAC-based and VCS-based regimens. Risk ratios (RRs) with 95% confidence intervals (CIs) were calculated from complete renal response event counts. Random-effects models (DerSimonian–Laird) were used as the primary synthesis because clinical heterogeneity was expected; common-effect estimates were examined as supportive analyses. Heterogeneity was quantified with I^2^, Cochran’s Q, and τ^2^. The TAC primary analysis pooled three clinically heterogeneous TAC-based strategies as an overall class-level synthesis, but this pooling was considered exploratory rather than evidence that TAC monotherapy and TAC + MMF multitarget therapy are interchangeable. Prespecified sensitivity analyses therefore separated TAC dual-therapy trials (Mok and Zheng) from the TAC + MMF multitarget trial (Liu) and examined comparator type. A formal Bucher TAC-versus-VCS comparison was not performed in the revised analysis because the TAC and VCS evidence sets do not share a sufficiently comparable common comparator and therefore do not satisfy the transitivity assumptions required for a valid adjusted indirect comparison.

Leave-one-out sensitivity analyses were performed for the TAC synthesis. Additional subgroup/sensitivity analyses examined TAC dual therapy versus TAC + MMF multitarget therapy and cyclophosphamide-comparator trials versus the MMF-comparator trial. For VCS, with only two eligible trials, sensitivity analysis consisted of presenting the individual trial estimates alongside the pooled estimate. All results are reported quantitatively in Section 3.7 rather than as narrative claims alone.

### 2.8. Small-Study Effects and Publication Bias

Formal statistical testing for funnel-plot asymmetry was not performed in the revised analysis. With only three TAC studies and two VCS studies, funnel plots and Egger’s regression are insufficiently powered and may be misleading [23]. Publication bias therefore could not be reliably assessed and was treated as an uncertainty of the evidence base rather than inferred from funnel-plot symmetry.

## 3. Results

### 3.1. Study Selection

**Figure 1:**
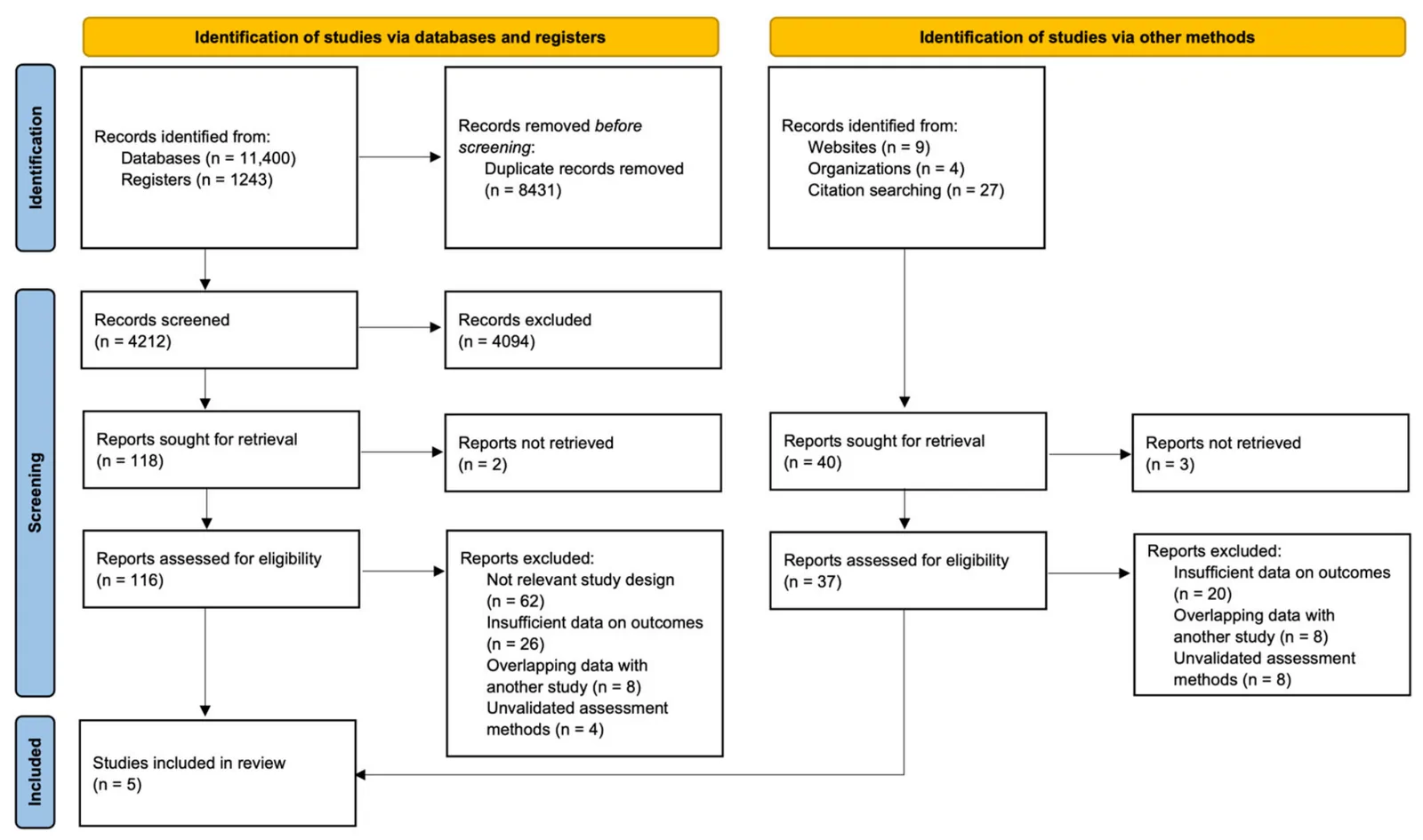
PRISMA flow diagram for the original search and study selection. The September 2026 search update identified one additional potentially relevant randomized trial that was excluded for not meeting the adult biopsy-confirmed eligibility criteria; the final quantitative synthesis remained five RCTs.

The original search identified 12,683 records. After duplicate removal and title/abstract screening, 153 reports underwent full-text assessment. Reports were excluded according to prespecified eligibility criteria, including non-randomized design, ineligible population or treatment setting, maintenance rather than induction therapy, and insufficient renal-response data to calculate or extract an effect estimate. Five RCTs ultimately met the inclusion criteria for quantitative synthesis, comprising three TAC trials and two VCS trials. Importantly, inclusion was not based on whether the treatment effect was favorable or statistically significant. The search was subsequently updated to September 2026; one additional randomized three-arm TAC/MMF/cyclophosphamide trial [24] was reviewed but excluded because it enrolled participants aged ≥10 years and did not require biopsy confirmation for all participants. The quantitative analytic set therefore remained five RCTs. The study-selection process is summarized in Figure 1.

### 3.2. Study Characteristics

The five included RCTs evaluated clinically distinct induction strategies. Mok et al. [18] compared TAC plus prednisolone with MMF plus prednisolone; TAC therefore functioned as the principal non-glucocorticoid immunosuppressive agent and should not be interpreted as an add-on CNI trial. Liu et al. [19] evaluated multitarget TAC + MMF plus corticosteroids against intravenous cyclophosphamide plus corticosteroids. Zheng et al. [20] compared TAC plus prednisone with intravenous cyclophosphamide plus prednisone. In contrast, AURA-LV [21] and AURORA-1 [22] evaluated VCS versus placebo on the same background of MMF and rapidly tapered low-dose corticosteroids. This difference in treatment architecture is central to interpretation: the TAC trials estimate effects of different TAC-based induction strategies against different active comparators, whereas the VCS trials estimate the incremental effect of adding VCS to a standardized MMF/glucocorticoid regimen.

The trials also used different analysis populations. Mok and Liu reported efficacy using the randomized trial populations; AURA-LV and AURORA-1 used intention-to-treat efficacy populations; Zheng randomized 314 participants, treated 299 (safety population), and reported the week-24 response analysis in a per-protocol efficacy set of 265 participants (141 TAC and 124 cyclophosphamide). These denominators were retained exactly for the event-based meta-analysis. Trial characteristics, treatment regimens, analysis populations, and response counts are summarized in Table 1.

### 3.3. Risk of Bias in Studies

**Figure 2:**
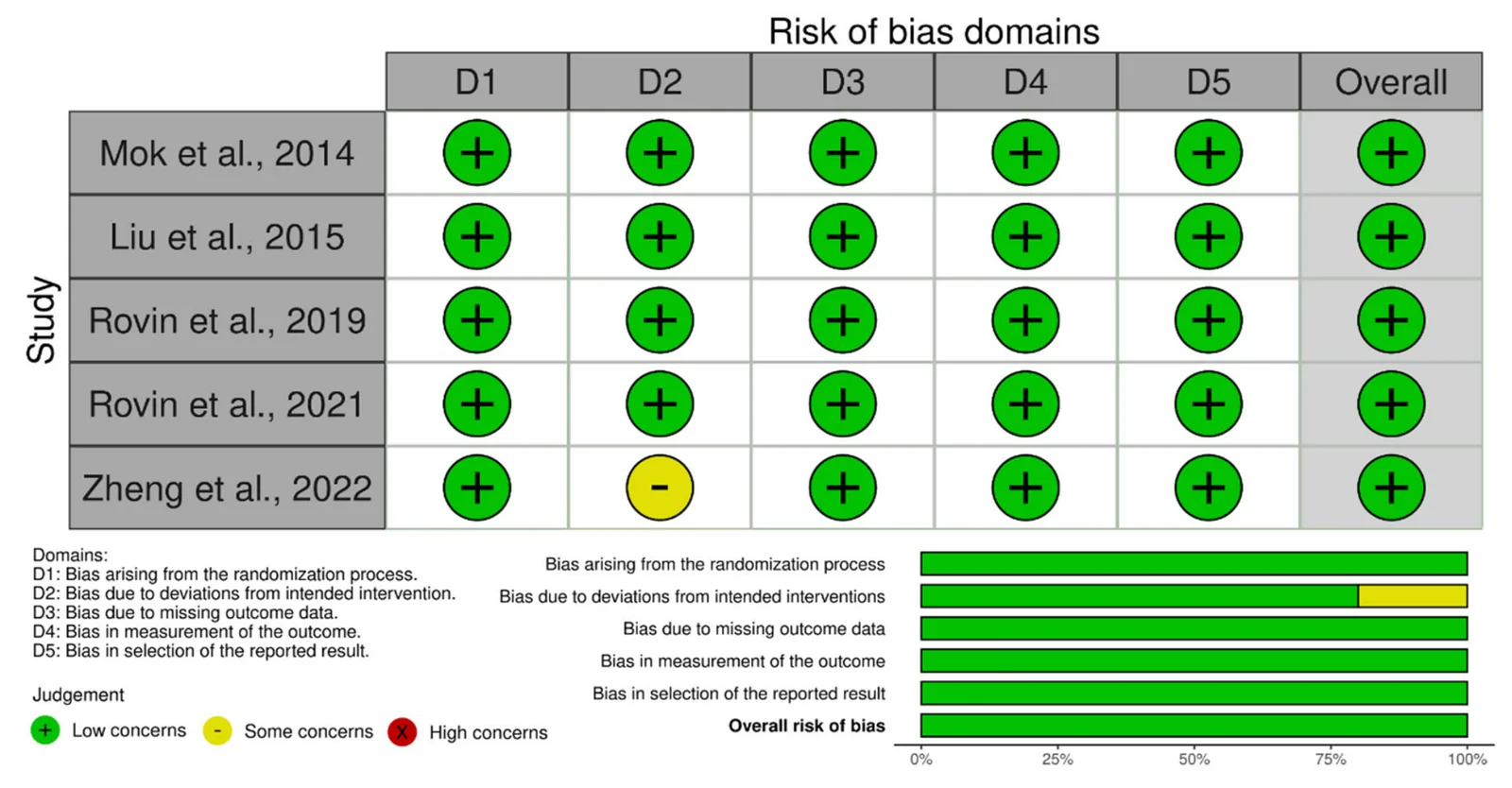
Application of the Cochrane RoB 2 tool to evaluate potential sources of bias across the included studies [18,19,20,21,22].

Methodological quality was evaluated using the Cochrane RoB 2 tool. Across the five included trials, the overall risk of bias was low. All studies employed appropriate randomization and allocation concealment procedures, and outcome assessors were blinded to treatment assignment. Four trials demonstrated low risk across all domains, while one TAC trial was rated as having some concerns related to deviations from intended interventions. No study exhibited a high risk of bias for missing data or selective outcome reporting. A visual summary of bias assessment across all domains is provided in Figure 2.

### 3.4. Results of Individual Studies

The primary efficacy outcome for the revised synthesis was complete renal response/remission at the prespecified induction endpoint. Trial definitions were not identical, particularly between earlier TAC studies and the VCS development program; therefore, estimates were interpreted as trial-defined complete renal response rather than as a perfectly uniform endpoint. The exact event counts and denominators used for each study are reported in Table 1.

### 3.5. Meta-Analysis

**Figure 3:**
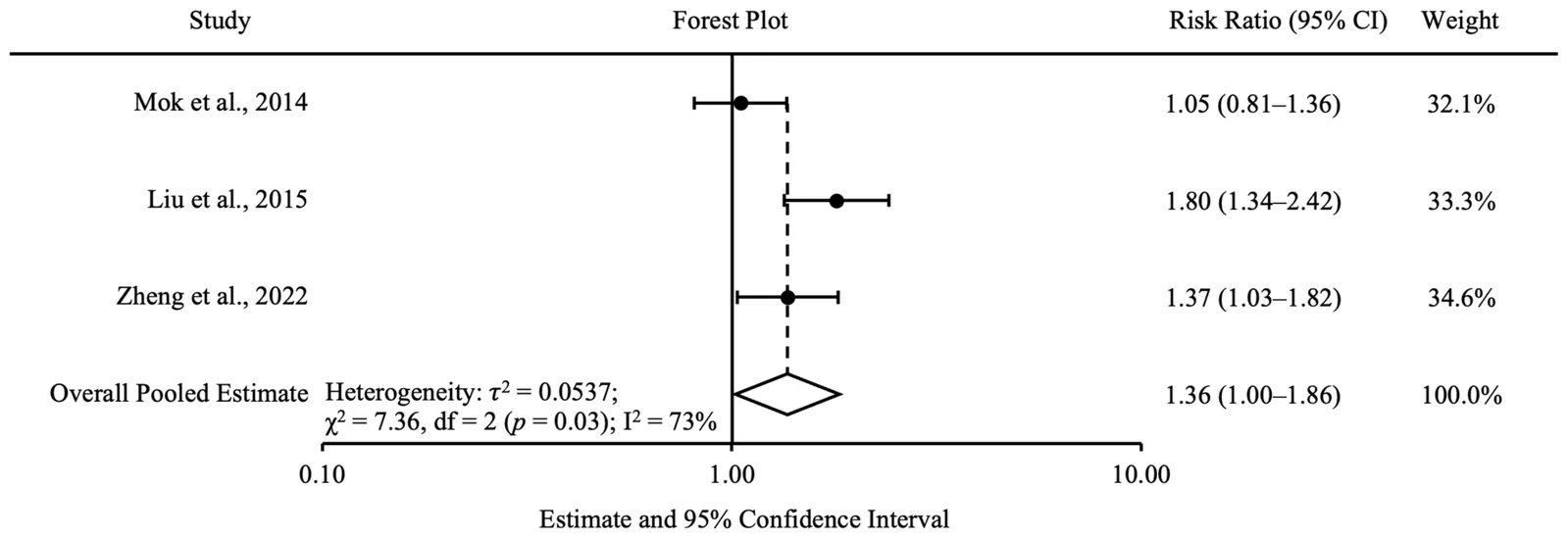
Forest plot of trial-defined complete renal response for TAC-based regimens versus their respective active comparators [18,19,20]. The pooled estimate combines clinically heterogeneous TAC strategies and should be interpreted together with the subgroup and leave-one-out analyses.

Across the three TAC-based RCTs, the random-effects pooled estimate for complete renal response was RR 1.36 (95% CI, 1.00–1.86), with substantial heterogeneity (I^2^ = 73%; τ^2^ ≈ 0.054). The individual effects were heterogeneous: Mok et al. RR 1.05 (95% CI, 0.81–1.36), Liu et al. RR 1.80 (95% CI, 1.34–2.42), and Zheng et al. RR 1.37 (95% CI, 1.03–1.82). Because Liu evaluated TAC + MMF multitarget therapy whereas Mok and Zheng evaluated TAC-based dual therapy, the pooled TAC estimate should be interpreted as a broad synthesis of TAC-based strategies rather than as a single homogeneous treatment effect (Figure 3).

**Figure 4:**
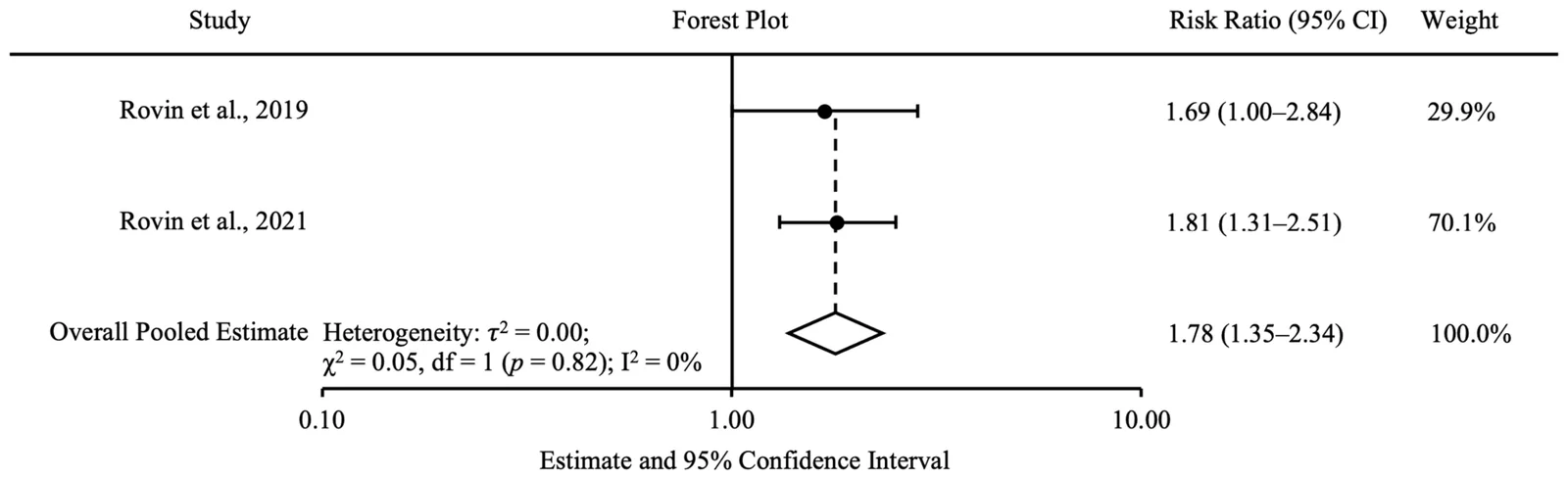
Forest plot of trial-defined complete renal response for VCS 23.7 mg twice daily versus placebo, with MMF and low-dose glucocorticoids administered in both groups [21,22].

For VCS, both eligible trials evaluated the same approved 23.7 mg twice-daily dose against placebo on a common MMF plus low-dose glucocorticoid background. The pooled complete renal response estimate was RR 1.78 (95% CI, 1.35–2.34), with no detected heterogeneity (I^2^ = 0%). Individual estimates were RR 1.69 (95% CI, 1.00–2.84) in AURA-LV and RR 1.81 (95% CI, 1.31–2.51) in AURORA-1. These estimates quantify the incremental effect of VCS within the standardized VCS trial regimen and should not be directly contrasted with the TAC pooled RR as if the comparator structures were equivalent (Figure 4).

No formal adjusted indirect TAC-versus-VCS comparison was retained in the revised analysis. The TAC evidence set used MMF or intravenous cyclophosphamide as active comparators and included both TAC dual therapy and TAC + MMF multitarget therapy, whereas the VCS evidence set used placebo on a standardized MMF plus low-dose glucocorticoid background. Because there is no sufficiently comparable shared comparator linking the two evidence sets, the transitivity and common-comparator assumptions required for a valid Bucher comparison were not satisfied. Accordingly, the previously reported exploratory TAC-versus-VCS RR was removed, and no claim of comparative efficacy or equivalence was made.

### 3.6. Small-Study Effects and Publication Bias

Publication bias could not be reliably assessed because only three TAC trials and two VCS trials were available. In accordance with standard meta-analytic guidance [23], Egger’s regression and funnel-plot symmetry were not interpreted because these methods have very low power with so few studies.

### 3.7. Sensitivity Analysis

Leave-one-out analysis demonstrated that the TAC pooled result was sensitive to study composition. Excluding Mok et al. yielded RR 1.57 (95% CI, 1.19–2.06; I^2^ = 42%). Excluding Liu et al., the TAC + MMF multitarget trial yielded RR 1.19 (95% CI, 0.92–1.54; I^2^ = 45%), indicating that the pooled statistical significance was not robust for removal of the multitarget trial. Excluding Zheng et al. yielded RR 1.37 (95% CI, 0.81–2.33; I^2^ = 86%). Thus, the primary TAC estimate should not be described as stable due to the exclusion of any single study.

When the two TAC dual-therapy trials (Mok and Zheng) were pooled without the multitarget trial, the random-effects estimate was RR 1.19 (95% CI, 0.92–1.54; I^2^ = 45%). Conversely, the two cyclophosphamide-comparator TAC trials (Liu and Zheng) yielded RR 1.57 (95% CI, 1.19–2.06; I^2^ = 42%); however, these trials still differed because one used TAC + MMF multitarget therapy and the other TAC without MMF. With only two VCS studies, leave-one-out analysis reduced the individual trial estimates: AURA-LV RR 1.69 (95% CI, 1.00–2.84) and AURORA-1 RR 1.81 (95% CI, 1.31–2.51). These analyses reinforce that the VCS evidence is more protocol-consistent, whereas the TAC pooled estimate is sensitive to regimen composition (Table 2).

Overall, sensitivity analyses do not support treating TAC monotherapy/dual therapy and TAC + MMF multitarget therapy as a single interchangeable intervention. The combined TAC meta-analysis is retained only as an exploratory summary of TAC-based induction strategies, with the dual-therapy and comparator-specific analyses providing the more clinically interpretable context.

### 3.8. Safety Analysis

Safety was summarized descriptively because the included trials used different comparators, follow-up periods, adverse-event definitions, and reporting thresholds; therefore, a pooled safety RR across all studies was not considered clinically appropriate. In Mok et al., major infections occurred in 4/74 TAC-treated patients (5.4%) and 7/76 MMF-treated patients (9.2%). In Liu et al., any adverse event occurred in 91/181 patients (50.3%) receiving TAC + MMF multitarget therapy and 95/181 patients (52.5%) receiving cyclophosphamide. In Zheng et al., serious treatment-emergent adverse events occurred in 29/157 TAC-treated patients (18.5%) and 35/142 cyclophosphamide-treated patients (24.6%), with serious study drug-related infections in 14/157 (8.9%) and 23/142 patients (16.2%), respectively. In AURA-LV, serious adverse events occurred in 25/89 patients (28.1%) receiving low-dose VCS and 17/88 (19.3%) receiving placebo; deaths were more frequent in the low-dose VCS group in this phase 2 trial (10/89 vs. 4/88). In AURORA-1, serious adverse events were balanced (37/178 [21%] with VCS vs. 38/178 [21%] with placebo), and deaths occurred in one VCS-treated patient and five placebo-treated patients. Because these safety endpoints were not uniformly defined or reported, they are presented as structured trial-level data rather than pooled comparative estimates (Table 3).

## 4. Discussion

### 4.1. Summary of Key Findings

This systematic review and meta-analysis separately synthesized randomized evidence for TAC-based and VCS-based induction regimens in adults with active LN. TAC-based regimens were associated with higher complete renal response relative to their respective active comparators in the three-study pooled analysis, but heterogeneity was substantial and the estimate was sensitive to inclusion of the Liu multitarget trial. VCS showed a consistent benefit when added to a standardized MMF plus low-dose glucocorticoid background in two placebo-controlled trials. These results should be interpreted within each evidence set; they do not establish relative efficacy between TAC and VCS.

Although class V lupus nephritis was represented in several included trials, class-specific analyses were limited by small subgroup sizes and were not the primary focus of these studies. Some tacrolimus trials reported numerically favorable responses in class V disease, and the voclosporin trials also included pure and mixed-class V populations; however, these subgroup findings were imprecise and should be considered hypothesis-generating. Accordingly, the available evidence does not permit firm conclusions regarding treatment effects of TAC- or VCS-based regimens specifically in pure class V lupus nephritis.

### 4.2. Proposed Mechanisms for Tacrolimus and Voclosporin in LN

The beneficial effects observed in this analysis may be explained by the shared and distinct pharmacologic mechanisms of TAC and VCS. Both drugs are potent CNIs, acting by binding to immunophilins, thereby inhibiting calcineurin phosphatase activity [25,26]. This prevents dephosphorylation of NFAT, a transcription factor essential for IL-2 gene transcription and T-lymphocyte activation [27]. The suppression of IL-2 production leads to diminished proliferation and activation of T-helper cells, reducing downstream B-cell stimulation and subsequent autoantibody production [28]. In LN, where immune complex deposition in the glomeruli and complement activation drive inflammation and tissue damage, dampening T-cell-mediated immune responses addresses one of the central pathogenic mechanisms.

Beyond these immunologic effects, CNIs also exert direct renal benefits that may contribute to the observed clinical responses. Both TAC and VCS have been shown to stabilize the podocyte actin cytoskeleton, thereby reducing proteinuria independent of their immunosuppressive activity [29,30]. Podocyte injury is a key feature of LN, particularly in classes III, IV, and V, where disruption of the glomerular filtration barrier contributes to ongoing protein loss and progressive renal injury [31,32]. By protecting podocyte structure and function, CNIs may provide a rapid antiproteinuric effect, which not only improves renal outcomes but also serves as an early marker of therapeutic efficacy. This dual mechanism of immune modulation and direct podocyte protection may explain the speed and magnitude of renal response reported in the included trials.

**Figure 5:**
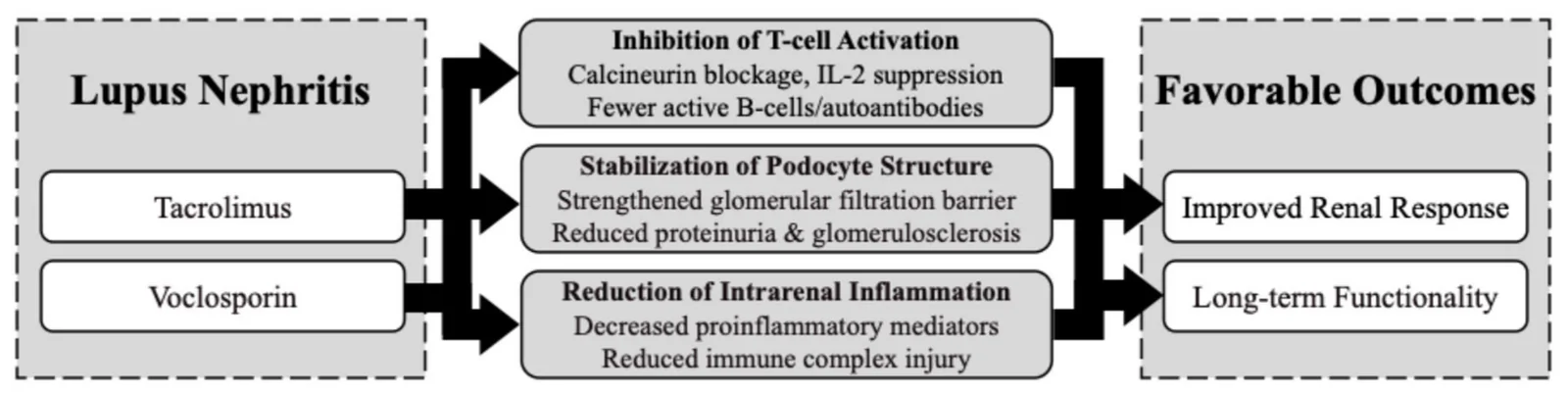
Proposed mechanisms underlying the association between TAC and VCS treatment and favorable renal outcomes in LN. CNIs may improve renal response through suppression of calcineurin–NFAT signaling, reduced T-cell activation and cytokine production, decreased downstream B-cell stimulation and autoantibody production, and stabilization of podocyte structure and function. These mechanisms may collectively reduce renal inflammation, proteinuria, and progressive glomerular injury.

While TAC has long been used in both transplant medicine and autoimmune nephropathies, VCS represents a structurally modified analog of cyclosporine designed to optimize potency, stability, and metabolic profile [33]. The addition of a functional group at the amino acid-1 residue in the structure of VCSs improves binding affinity to calcineurin and alters its metabolism, allowing for more predictable pharmacokinetics and reduced intra- and inter-patient variability compared to cyclosporine [34,35]. This may partly explain the more consistent outcomes seen across VCS trials. Furthermore, VCS does not require therapeutic drug monitoring, compared to TAC, which could translate into improved adherence and broader applicability in clinical practice, particularly in resource-limited settings where drug-level monitoring is not routinely available. Collectively, these immunologic, podocyte-related, and pharmacokinetic mechanisms provide biologic support for the renal response benefits observed across studies (Figure 5).

### 4.3. Implications for Clinical Practice

The clinical implication of this review is that both TAC-based and VCS-based strategies have randomized evidence supporting renal response in active LN, but the evidence structures are not directly comparable. The TAC literature spans distinct treatment strategies and active comparators, whereas VCS was studied as an add-on to a standardized MMF plus low-dose glucocorticoid regimen. Consequently, treatment selection should not be based on the numerical difference between the TAC and VCS pooled RRs. Instead, clinicians should consider the specific regimen supported by each trial, safety profile, monitoring burden, comorbidities, cost and access, and current guideline recommendations [11,36]. Head-to-head comparative trials remain necessary to determine whether one CNI strategy offers superior efficacy or safety.

### 4.4. Strengths and Limitations

This study has several strengths. It focuses on randomized evidence, uses exact event counts and trial-reported analysis populations, separates TAC and VCS syntheses, and explicitly examines the clinical heterogeneity of TAC regimens. The revised analysis also reports quantitative leave-one-out and subgroup results rather than relying on narrative assertions, and it summarizes safety outcomes in a structured trial-level format.

Several limitations should be acknowledged. First, the evidence of TAC is clinically heterogeneous: Mok and Zheng evaluated TAC-based dual therapy, whereas Liu evaluated TAC + MMF multitarget therapy. Pooling these studies therefore provides only an exploratory class-level estimate, and sensitivity analysis excluding Liu yielded RR 1.19 (95% CI, 0.92–1.54), demonstrating that the primary TAC result is sensitive to regimen composition. Second, the comparator structure differs fundamentally between the TAC and VCS evidence sets; TAC was compared with MMF or cyclophosphamide, while VCS was compared with placebo against a background of MMF and low-dose glucocorticoids. This prevents a valid formal Bucher comparison and precludes claims of relative efficacy.

Third, analysis populations were not uniform. In particular, Zheng reported efficacy using a per-protocol population while safety was assessed using the treated population; the revised manuscript therefore reports exact denominators for each analysis. Fourth, complete renal response definitions and assessment times differed across trials, ranging from 24 weeks to 52 weeks. Fifth, because this focused review required renal-response outcomes to be reported in a form permitting extraction or calculation of an effect estimate, selective outcome-reporting bias cannot be excluded. Studies were not selected according to the direction or statistical significance of the treatment effect; otherwise relevant trials that measured but did not report renal response in an extractable form would not have contributed to the synthesis. The findings should therefore be interpreted as a synthesis of the available randomized evidence with quantifiable renal-response reporting rather than as evidence encompassing every trial in which TAC or VCS may have been studied. Sixth, most TAC evidence arose from Asian populations, which may limit generalizability. Finally, safety outcomes were reported using nonuniform definitions and thresholds, so they were summarized descriptively rather than pooled.

Publication bias also remains uncertain. With only three TAC and two VCS trials, formal tests for funnel-plot asymmetry have inadequate power [23]; Egger’s tests and visual funnel-plot claims were removed from the revised analysis. The small number of trials also limits subgroup analyses by histologic class, baseline proteinuria, ethnicity, and other potential effect modifiers.

### 4.5. Future Directions

The mechanistic overlap between TAC and VCS raises intriguing questions for future research. Direct comparative trials are needed to establish whether the pharmacokinetic advantages of VCS translate into superior long-term efficacy, safety, or adherence compared to TAC. Such trials should include a uniform background therapy as outlined by recent ACR guidelines for management of LN [11], diverse patient populations including different ethnicities, extended follow-up, and comprehensive assessment of renal and extrarenal SLE outcomes. Given the immunologic complexity of LN, biomarker-driven approaches may also help identify the patients most likely to benefit from CNI therapy. For example, baseline levels of podocyte injury markers, complement activation products, or T-cell activation signatures could potentially predict treatment response and inform personalized therapy selection.

Future studies should also address the optimal duration of CNI therapy in LN. While early initiation may be key to achieving rapid remission, prolonged exposure increases the risk of cumulative toxicity. Determining whether CNIs can be safely tapered or discontinued after achieving remission and identifying predictors of relapse is essential for minimizing long-term harm. Additionally, exploration of combination strategies, such as pairing CNIs with biologic agents targeting B-cell activation (e.g., belimumab) or type I interferon signaling, could further improve outcomes, particularly in refractory disease.

Another important avenue for investigation is the impact of CNIs on health-related quality of life, treatment satisfaction, and medication adherence. The burdens of polypharmacy, frequent monitoring, and adverse effects can be substantial for patients with LN, and differences in tolerability or ease of use between TAC and VCS may have meaningful implications for real-world effectiveness. Similarly, cost-effectiveness analyses comparing the two agents in different healthcare settings would provide valuable guidance for policymakers and clinicians, especially in regions where drug costs and monitoring infrastructure vary widely. This is important taking into consideration that VCS is FDA-approved for the management of LN, while TAC is not.

## 5. Conclusions

This systematic review and meta-analysis found that TAC-based and VCS-based induction regimens each improved complete renal response relative to the comparators used in their respective randomized trials. The TAC evidence was heterogeneous and sensitive to inclusion of the TAC + MMF multitarget trial, whereas the VCS trials used a more standardized MMF plus low-dose glucocorticoid background. Because the TAC and VCS evidence sets differ in treatment architecture, comparators, populations, and outcome definitions, the available randomized evidence does not support a reliable indirect estimate of comparative efficacy between TAC and VCS. Future head-to-head trials with standardized background therapy, efficacy definitions, safety ascertainment, and longer follow-ups are needed to guide comparative treatment selection.

## Figures and Tables

**Table 1:** Characteristics of included studies.

Study	Study Design	Sample Size (Treatment: Control)	Age, Years	Sex (%)	Treatment Arms	Trial Duration
Mok et al., 2014 [18]	Randomized controlled trial	150 (74:76)	35.5 (12.8)	M: 8.0; F: 92.0	TAC + prednisolone vs. MMF + prednisolone	26 weeks
Liu et al., 2015 [19]	Randomized controlled trial	362 (181:181)	31.9 (2.8)	M: 9.1; F: 90.9	TAC + MMF + corticosteroids vs. IVCY + corticosteroids	24 weeks
Rovin et al., 2019 [21]	Randomized controlled trial	265 (177:88)	31.7 (10.5)	M: 13.2; F: 86.8	VCS (23.7 or 39.5 mg BID) + MMF + low-dose corticosteroids vs. placebo + MMF + low-dose corticosteroids	48 weeks *
Rovin et al., 2021 [22]	Randomized controlled trial	357 (179:178)	32.3 (9.1)	M: 12.3; F: 87.7	VCS 23.7 mg BID + MMF + low-dose corticosteroids vs. placebo + MMF + low-dose corticosteroids	52 weeks
Zheng et al., 2022 [20]	Randomized controlled trial	299 (157:142)	34.2 (9.5)	M: 12.4; F: 87.6	TAC + prednisone vs. IVCY + prednisone	24 weeks

Abbreviations: MMF = mycophenolate mofetil; TAC = tacrolimus; VCS = voclosporin; IVCY = intravenous cyclophosphamide; BID = twice daily. Age is presented as mean (SD). * In AURA-LV (Rovin et al., 2019 [21]), the primary renal response endpoint was assessed at 24 weeks, although patients were treated and followed for 48 weeks.

**Table 2:** Quantitative sensitivity and subgroup analyses for complete renal response.

Analysis	Studies	RR (95% CI)	I^2^
TAC primary random-effects	Mok + Liu + Zheng	1.36 (1.00–1.86)	73%
Leave-one-out: exclude Mok	Liu + Zheng	1.57 (1.19–2.06)	42%
Leave-one-out: exclude Liu multitarget	Mok + Zheng	1.19 (0.92–1.54)	45%
Leave-one-out: exclude Zheng	Mok + Liu	1.37 (0.81–2.33)	86%
TAC dual therapy only	Mok + Zheng	1.19 (0.92–1.54)	45%
TAC trials with IVCY comparator	Liu + Zheng	1.57 (1.19–2.06)	42%
VCS pooled	AURA-LV + AURORA-1	1.78 (1.35–2.34)	0%

Abbreviations: TAC = tacrolimus; VCS = voclosporin; IVCY = intravenous cyclophosphamide; RR = risk ratio; CI = confidence interval.

**Table 3:** Selected safety outcomes reported in the included randomized controlled trials.

Study	Intervention Regimen	Comparator Regimen	Safety Population (Intervention/Comparator)	Selected Safety Outcome	Intervention vs. Comparator
Mok et al., 2014 [18]	TAC + prednisolone	MMF + prednisolone	74/76	Major infections	4/74 (5.4%) vs. 7/76 (9.2%)
Liu et al., 2015 [19]	TAC + MMF + corticosteroids	IVCY + corticosteroids	181/181	Any adverse event	91/181 (50.3%) vs. 95/181 (52.5%)
Rovin et al., 2019 (AURA-LV) [21]	VCS 23.7 mg twice daily + MMF + low-dose corticosteroids	Placebo + MMF + low-dose corticosteroids	89/88 *	Serious adverse events	25/89 (28.1%) vs. 17/88 (19.3%)
Rovin et al., 2021 (AURORA-1) [22]	VCS 23.7 mg twice daily + MMF + low-dose corticosteroids	Placebo + MMF + low-dose corticosteroids	179/178	Serious adverse events	Approximately 21% vs. 21%
Zheng et al., 2022 [20]	TAC + prednisone	IVCY + prednisone	157/142	Serious treatment-emergent adverse events	29/157 (18.5%) vs. 35/142 (24.6%)
Zheng et al., 2022 [20]	TAC + prednisone	IVCY + prednisone	157/142	Serious infections related to study treatment	14/157 (8.9%) vs. 23/142 (16.2%)

Abbreviations: MMF = mycophenolate mofetil; TAC = tacrolimus; VCS = voclosporin; IVCY = intravenous cyclophosphamide. * For AURA-LV, the table presents the low-dose voclosporin 23.7 mg twice-daily arm used in the efficacy synthesis rather than combining both voclosporin dose groups.

## Data Availability

This review was not registered and no new primary data was generated in support of this study. All analyzed data are derived from previously published studies and are listed in the references.

## Supplementary Materials

The following supporting information can be downloaded at: https://www.mdpi.com/article/10.3390/jcm15197607/s1, Supplementary File S1: PRISMA 2020 Checklist.
